# Advanced practice nurse intervention versus usual care for hypertension control: study protocol for an open-label randomized controlled trial

**DOI:** 10.1186/s13063-023-07437-3

**Published:** 2023-07-03

**Authors:** Juliette Vay-Demouy, Alexandre Cinaud, Nathan Malka, Baptiste Mion, Sandrine Kretz, Hélène Lelong, Jacques Blacher

**Affiliations:** Diagnosis and Therapeutic Center, Hôtel-Dieu University Hospital, Assistance Publique-Hôpitaux de Paris, Paris Cité University, Paris, 75004 France

**Keywords:** Advanced practice nurse, Hypertension, Control, Blood pressure, Protocol study

## Abstract

**Background:**

Hypertension is the most frequent chronic pathology in France and in the world. It is one of the main modifiable cardiovascular risk factors. In France, 50% of treated hypertensives are uncontrolled and only 30% of treated patients are fully adherent to their antihypertensive treatment. Poor adherence to drug treatments is considered as one of the main causes of non-control of hypertension. Since 2018, a new profession has entered the French healthcare system: advanced practice nurses (APN). They have many broad-based skills, at the interface of nursing and medical exercises. The purpose of this study is to assess the impact of an APN intervention versus usual care on hypertension control.

**Methods:**

The study will take place at the Hôtel-Dieu University Hospital, Paris, France, as prospective, open-label, controlled, randomized 1-to-1, monocentric, and superiority trial. The participants will be recruited during day hospitalization for cardiovascular assessment in the context of their hypertension management. Patients will be divided into two groups: a “usual care” group which will continue traditional follow-up (day hospitalization followed by consultation with a medical doctor (MD) within approximately 2–12 months) and an “intervention” group which will meet an APN between the day hospitalization and the MD consultation. Participants will be monitored until 12 months after the day hospitalization, depending on their last follow-up study appointment (MD consultation). The primary outcome is the rate of controlled BP (BP < 140/90 mmHg in office BP measurement) in each group. The hypothesis formulated is that an individual APN intervention, included in usual hypertension management, improves hypertension control.

**Discussion:**

This innovative study will be the first in France where APNs are beginning to be established in the healthcare system. It will provide an objective look at this new profession and the impact it can have in the framework of global management of hypertension.

**Trial registration:**

ClinicalTrials.gov NCT0448249. Registered on June 24, 2020.

## Background

Hypertension is defined by blood pressure (BP) ≥ 140/90 mmHg in office BP measurement after repeated consultation and/or BP ≥ 135/85 mmHg by home BP monitoring regardless of antihypertensive drug use [1–4]. It is the most prevalent chronic pathology in France and in the world [3]. It is the main modifiable cardiovascular, cerebrovascular, renal, and neurodegenerative risk factor and accounts for 13% of mortality worldwide (more than 10 million deaths) [5, 6].

In France, in 2015, the prevalence of hypertension was 31.3% of people aged between 18 and 74, according to ESTEBAN, a cross-sectional study implemented by Santé Publique France, a national public health agency, from 2014 to 2016 on a representative sample of the French population. Among these hypertensive adults, 43.7% were unaware of hypertension, and only 50.3% of those treated were controlled. Only 33.6% of treated hypertensive patients were considered adherent [7]. This rate is similar for all the main cardiovascular risk factors, such as type 2 diabetes and dyslipidemia [8, 9].

Nearly 200 countries, including France, have joined the World Health Organization Global Plan of Action for the Control of Non-Communicable Diseases 2013–2020. Reducing the prevalence of hypertension is one of the targets of this project through the implementation of policies promoting a healthier lifestyle: weight control, increased physical activities, healthy diet, alcohol consumption reduction, and tobacco fight strategies [10].

Poor adherence to drug treatments is considered as one of the main causes of uncontrolled hypertension. Non-adherence can affect up to 80% of hypertensive patients [1] and is often defined by taking less than 80% of the days covered by prescribed medication. Early discontinuation of treatments, suboptimal daily use of the prescribed regimens, and difficulties in adapting a healthy lifestyle according to the recommendations are the main aspects of non-adherence [1, 2]. Lifestyle changes are known to prevent or delay the onset of hypertension and cardiovascular complications. Recent recommendations including the 2018 Guidelines for the Management of Arterial Hypertensions of the European Society of Hypertension promote healthy behaviors [1–3].

These deficiencies in therapeutic adherence and disease objective control can be explained by a person’s inability to take his or her treatment, including socioeconomic constraints, hostility, depression, and anxiety. Factors associated with poor adherence include low educational level and social isolation [3, 11–16]. Widely spaced consultations of short duration and lack of time for health education and for close monitoring are deleterious for long-term hypertension control and therapeutic alliance development [17].

Health education for hypertension management is considered as one of the best ways to prevent cardiovascular complications [18]. Hypertension can be asymptomatic; it is necessary to inform patients about its possible complications so as to involve them in monitoring [4]. In addition, the chronic aspect of hypertension complicates its management. It can be difficult for patients to admit their vulnerability, to take long-term daily treatment, and to conduct adequate monitoring. For a better hypertension control, it seems necessary to improve management and to reinforce the therapeutic alliance. Better communication between caregivers and patients is conducive to accurate diagnosis, treatment choice, adherence, and patient satisfaction [19]. It is possible to improve this aspect of comprehensive care through personalized, in-depth health education.

Since July 2018, a new profession has emerged in the French healthcare system: advanced practice nurses (APN). This profession has existed for several decades in other countries such as Canada, the USA, Australia, and the UK. These countries have already established this profession in their healthcare system, even institutional several protocols related to advanced nursing practice. The International Council of Nurses defined APN in 2002 as follows: “An APN is a State-certified or certified nurse who has acquired the theoretical knowledge, the know-how necessary for making complex decisions, as well as the clinical skills essential to the advanced practice of his profession, advanced practice whose characteristics are determined by the context in which the nurse will be authorized to practice” [20]. This definition identifies the bases of this exercise while leaving each country free to adapt this practice according to the context and its needs in terms of health [21].

In France, APN training and exercise are based on National Decree No. 2018-629 [22]. APNs have many broad-based skills, at the interface of nursing and medical practice. These new skills allow them not only to prescribe and interpret additional exams, but also to renew prescriptions introduced beforehand by a medical doctor (MD). APNs can monitor patients suffering from chronic pathologies, such as hypertension, alternating with MDs in the framework of a commonly established organizational protocol.

This new profession in France has appeared in a context marked by several public health challenges, such as a decreased number of MDs and increased chronic pathologies over time with the aging of the population.

This article introduces the protocol of a study aimed at assessing the impact of an APN intervention, as part of usual care, on hypertension control.

## Methods

### Conception of the study

The study was conceived by a team of doctors and an APN working at the Diagnosis and Therapeutic Center of the Hôtel-Dieu University Hospital, Assistance Publique – Hôpitaux de Paris, France. This department is one of the labeled European centers of excellence for hypertension.

A pilot study lasting several months was carried out in the department in 2022. It allowed us to develop the protocol, thanks largely to the patients involved, whose opinions were solicited. It has not been published.

The protocol has been reviewed following the SPIRIT guidelines.

### Study design

**Fig. 1:**
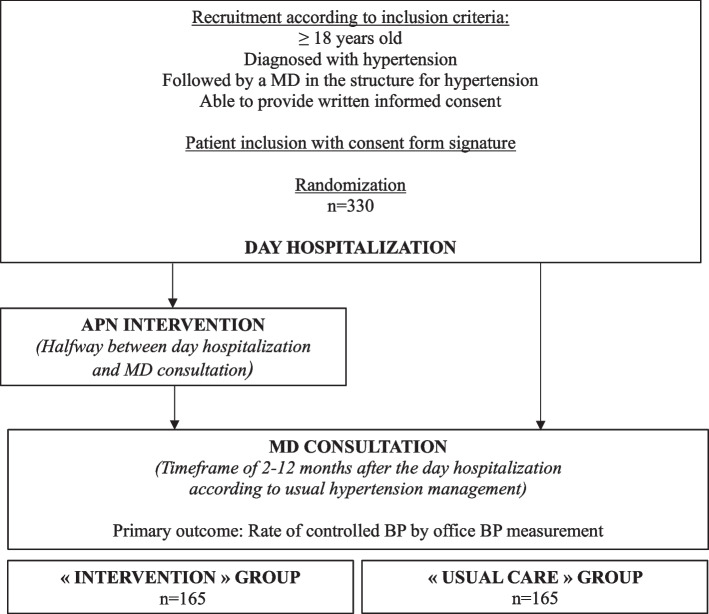
Study process

This study will be a prospective, open-label, randomized 1-to-1, controlled, and monocentric superiority trial, conducted at the Diagnosis and Therapeutic Center of the Hôtel-Dieu University Hospital, Assistance Publique – Hôpitaux de Paris, France (Fig. 1).

Recruitment should start during spring 2023 and last approximately 1 year.

### Participants

The patients will be recruited during their visit to the day hospitalization section of the Diagnosis and Therapeutic Center of Hôtel-Dieu University Hospital, for hypertension-related complications and cardiovascular risk assessment as part of their hypertension management.

Participants will be eligible if they are at least 18 years of age, diagnosed with hypertension (treated or untreated), followed by a MD in the structure for hypertension management, and able to provide written informed consent. Hypertension is defined by BP ≥ 140/90 mmHg by office BP measurement and/or BP ≥ 135/85 mmHg by home BP monitoring.

The non-inclusion criteria will be patients under 18 years old, APN follow-up prior to day hospitalization, pregnancy, guardianship/curatorship, or inability to give free informed consent.

Written information and oral explanations will be given to patients. To complete their inclusion in the study, free informed and written consent will be collected from each participant during the day of hospitalization.

The dropout criteria will be an acute cardiovascular event during the study period and/or therapeutic adjustment of hypertension treatment by another health professional than the APN or MD referent during the study period and/or refusal to proceed in the study.

### Relevant concomitant care and interventions during the trial

During the period of participation in the research, patients will not be allowed to participate in another research protocol involving human beings without having discussed it with the physician following him or her in the context of the research. Nevertheless, patients may participate in other non-interventional research.

At the end of the patients’ participation, no exclusion period will be required.

### Aims

We assume that individual APN intervention, as part of the usual care management of hypertension, can improve hypertension control.

Our primary objective will be:

1. To compare the rate of controlled BP in “intervention” and “usual care” groups

Our secondary objectives will be:

1. To assess adherence to the home BP monitoring protocol
2. To assess therapeutic adjustments of antihypertensive drugs and their indications (efficacy and/or tolerance) during the APN intervention
3. To describe the evolution of BP control between the day of hospitalization and the MD consultation in each group.

### Outcomes

All outcomes will be evaluated on a timeframe of 2–12 months depending on the period between the day of hospitalization (inclusion visit) and the MD consultation (final visit of the study).

The primary outcome will be:

1. Rate of controlled BP in MD consultation by office BP measurement

According to the European and International guidelines, the protocol for office BP measurement will be 5 min rest, 3 measurements at 1-min intervals in a supine position followed by 3 standing measurements at 1-min intervals for orthostatic hypotension test [1–4]. Unattended office BP measurement will be conducted by a supervisor (usually a nurse), who will explain the protocol to the patient before letting him rest in a quiet room: an automatic sphygmomanometer that will print the measurement statement at the end of the monitoring session. BP level will be estimated by the average of the last two BP measures in the supine position. BP will be considered as controlled with systolic BP < 140 mmHg and diastolic BP < 90 mmHg [1–4].

As a reminder, only the BP level by office BP measurement during the MD consultation (final visit of the study) will constitute the primary outcome.

The secondary outcomes will be:

1. Rate and quality of home BP monitoring brought to MD consultation.

According to the 2020 International Society of Hypertension (ISH) Global Hypertension Practice Guidelines, the protocol for home BP monitoring will be 3-day monitoring with a cycle of 3 measurements every morning and every evening at 1-min intervals, in a sitting position, after 5 min of rest and before meals [1]. Quality will be assessed by the number of measurements (18 measurements over 3 days).

2) Therapeutic adjustments (and their indication(s)) will be collected in the medical file due to the traceability of the prescriptions of antihypertensive treatment and the APN intervention report.
3) Difference in rates of controlled BP between day hospitalization and MD consultation in each group. Note that BP level during day hospitalization is measured with the same protocol as during MD consultation.

### Sample size

For primary outcome measurement, according to the preliminary results of our pilot study, we estimated the number of patients to be included in our trial. We made the hypothesis of 50% and 65% of controlled BP in the “usual care” and “intervention” groups, respectively, equivalent to a 30% increase in the rate of controlled hypertensive participants in the “intervention” group (corresponding to a 15% absolute increase or a 30% relative increase in the controlled BP rates). Assuming an equal number of patients in the two groups, a chi-squared test with a 2-sided significance level of 5%, and a power of 85%, it will be necessary to recruit 300 patients (150 per arm). With a 10% margin of loss to follow-up, 330 patients will need to be recruited (165 per arm).

Sample size calculation was performed with the SAS software (version 9.4; Institute Carry, NC).

### Recruitment

Recruitment will be conducted during the day of hospitalization, by the residents of the unit, according to the recommendations of the study coordinator.

The residents will explain the study protocol to eligible patients, propose to them to participate and give them an information notice and a consent form to sign. Patients will have to indicate if they consent to participate or not (+/− sign the form) before the end of the day of hospitalization.

After having collected the signed consent form, the residents will give the consent form to the APN/MD to countersign before randomization at the end of the hospital day.

### Sequence generation

Block randomization will be conducted by Dr. Hélène Lelong, a MD in the unit trained in biostatistics, using the randbetween formula (0 to 100) on the Excel software for Windows, by blocks of variable size. The even numbers will constitute the “intervention” group and the odd numbers the “usual care” group. This method will ensure the homogeneity and unpredictability of the randomization during the study period. No stratification is scheduled.

The randomization list will then be copied onto a permanent registry. Allocation groups will be concealed in individual numbered opaque sealed envelopes (one per participant). The number and allocation for each envelope will be assigned according to the randomization list.

The individual sealed envelopes will be given to the day hospital nurses who will randomize participants. Nurses will never have access to the randomization list.

### Randomization

Day hospital nurses will randomize participants. They will be involved in hypertension management only in the day hospital, neither before nor after, and will have no interest in generating any bias in the randomization.

At the last moment in the day hospital (after patient inclusion and consent form signature), the nurse in charge of scheduling the follow-up appointments will randomize each participant through an individual numbered envelope mentioning the allocation group. According to the allocation group, the nurse will make the following appointments: APN and MD appointments for the “intervention group” or MD appointment only for the “usual care” group. The participants will go home just after receiving their next appointment(s).

Nurses will not be allowed to open the envelopes prior to the randomization or to change the order of the envelopes. Compliance with the randomization list will be verified by the number attributed to each envelope and anomalies will be identified if the randomization sequence has been violated.

### Allocation concealment

As a reminder, this study cannot be blinded because follow-up appointment(s) must be scheduled according to the allocation group.

Nevertheless, allocation concealment is preserved for as long as possible through the individual numbered opaque sealed envelopes and thanks to the nurses who will proceed to randomization as late as possible: just before scheduling the follow-up appointment(s) and sending the patient home.

The investigator and the statistician will never have access to the randomization list.

### Ethical compliance

The study is registered in the French National Agency for Medicines and Health Products Safety (No. 2020-A00158-31).

### Equipment

During the day hospitalization, a tensiometer will be given to all participants, whether they have one at home or not and regardless of their group, the objective being to avoid any measurement bias in home BP monitoring protocol.

The tensiometer meets the recommendations of the French Society of Arterial Hypertension [23].

### Home BP monitoring

A dedicated home BP monitoring sheet with oral and written explanations for home BP monitoring protocol will be given with the tensiometer to all participants during the day of hospitalization. Participants will be asked to perform only one home BP monitoring, a few days before their next follow-up consultation(s): MD consultation for all participants ± APN intervention according to the allocation group.

The home BP monitoring protocol is detailed above in the “Outcomes” section.

No interpretation of the home BP monitoring result will be requested from the participants. Nevertheless, they will be able to contact the unit if they are concerned.

The protocol will be explained again during the APN intervention for the “intervention” group if needed.

### The APN and associated competences

According to the national decree, an APN requires a nurse to have practiced for at least three years and to have obtained a master’s degree.

The APN must not only retain the skills specific to nursing, but also acquire other specific skills for the management of chronic pathologies, including hypertension.

The APN takes care of patients as part of the treatment of their stabilized chronic pathologies. The management of acute events and decompensation for chronic conditions are left to the MD.

Decree n° 2018-629 defines APN competences as “carrying out any clinical evaluation and conclusion act or any clinical and paraclinical surveillance act”; performing reference monitoring, prevention, and technical acts; and renewing medication [22, 24].

### APN intervention

The APN intervention will be scheduled halfway between the day of hospitalization and the MD consultation. The MD consultation will first be scheduled 2–12 months after the day of hospitalization according to usual hypertension management and the APN intervention will then be scheduled 1–6 months after the day of hospitalization according to the time of the MD consultation for the “intervention” group. For example, if the MD consultation is scheduled 8 months after the day of hospitalization, then the APN intervention will be scheduled 4 months after the day of hospitalization. We kept the minimum time for follow-up at 1 month because this is the minimum time needed to reassess the efficacy and tolerance of antihypertensive treatment in case of therapeutic change during the day hospital session.

The APN intervention will always take place in the hospital, at the consultation unit of the Diagnosis and Therapeutic Center of Hôtel-Dieu University Hospital, at the same location as the MD consultation.

**Fig. 2:**
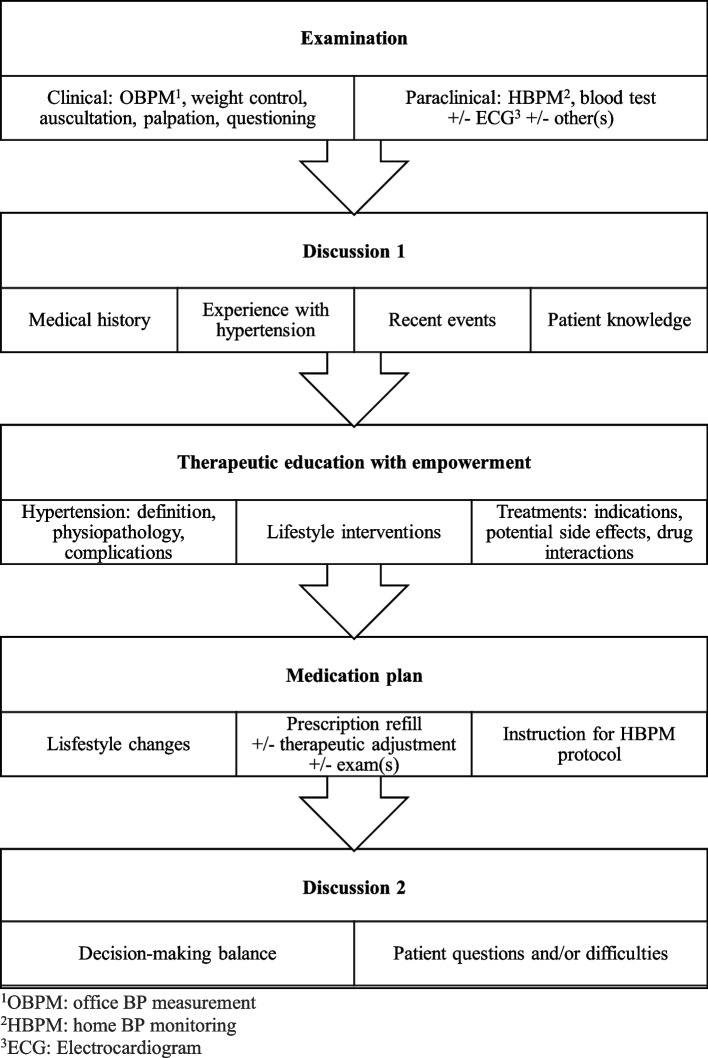
Plan for the APN intervention

The APN intervention will be divided into different stages: examination, discussions, therapeutic education with empowerment, and medication plan (Fig. 2).

The APN intervention will be performed by the same APN hypertension specialist and will last approximately 45 min to 1 h.

First, the APN will proceed to office BP measurement. The APN will explain the procedure for office BP measurement, check the correct functioning of the automatic sphygmomanometer, and install the patient in a quiet room to minimize unattended results. As described above, office BP will be measured according to the latest recommendations: 5 min of rest without speaking or smoking, 3 measurements at 1-min interval in a supine position followed by 3 measurements in a standing position for orthostatic hypotension test. These measurements will be performed with an oscillometric automatic sphygmomanometer. The retained office BP measurement will be the average of the last two measurements in the supine position [1, 2, 23]. This office BP measurement will be compared to the home BP monitoring measurement. While home BP monitoring is usually considered superior, according to its quality and taking into account potential masked or white coat hypertension, either office BP measurement or home BP monitoring will be preferred by the APN to evaluate the BP level and the efficacy of the antihypertensive drugs taken during the study [1, 2].

Through a clinical exam, the APN will then assess the general statement of health, efficacy, and potential side effects of antihypertensive treatment such as lower limb edema or cough, as well as clinical manifestations of hypertension or its complications such as headache, vertigo, syncope, impaired vision, rest or exertion chest pain, dyspnea, palpitations, intermittent claudication, edema, and cold extremities. The search for side effects will be completed, if necessary, by a cardio-pulmonary auscultation and/or an electrocardiogram (ECG). The APN will immediately refer clinically unstable patients to an MD.

The most recent blood tests will be checked to assess potential side effects of antihypertensive treatment such as dyskalemia or acute kidney injury and/or cardiovascular comorbidities such as diabetes or dyslipidemia according to the latest recommendations [25].

The APN will sum up the medical history, assess the patient’s knowledge and remind him or her of the definition, the pathophysiology, the chronic aspect of hypertension, and its possible complications.

The APN will then assess the lifestyle and advise the patient according to the latest recommendations [1–3, 23]:

- Salt consumption limitation (about 6 to 8 g a day)
- Weight reduction in the event of overweight or obesity to maintain a normal body mass index (between 18.5 and 24.9)
- Practice of regular physical activity of approximately 30 min a day (to be adapted according to the clinical condition of the patient)
- Alcohol consumption limitation to less than 14 units per week for men, 8 units per week for women
- Establishment of a diet rich in fiber and low in saturated fat
- Smoking cessation

Patients’ knowledge about drugs, their indications, and mechanisms will be evaluated and updated if necessary. To estimate the overall risk of drug interaction, the APN will ask the patient about a possible additional prescription or self-medication.

While posology adaptation in the event of side effects or uncontrolled hypertension will be carried out when necessary, the APN will refer the patient to the MD if an introduction of a new drug is needed.

The APN will propose to the patient a decision-making balance between benefits and risks, the objective being to encourage the patient to improve therapeutic adherence, to promote a healthy lifestyle, and to establish in coordination with the patient a treatment plan to improve adherence.

The end of the patient interview will be dedicated to unmentioned questions or subjects, requiring in-depth consideration.

The APN will give the patient a new home BP monitoring sheet with a reminder of the procedure to follow. The patient will be required to bring the results to his or her next MD consultation.

The APN intervention report will be digitally available in the patient’s electronic medical file.

If a participant does not show up for the APN intervention, we will call him/her to schedule another appointment in the limit of the data collection timeframe. If he/she still does not show up, the allocation group will remain the same. The rate of participants not having shown up for the APN intervention will be mentioned in the result.

### MD consultation

**Fig. 3:**
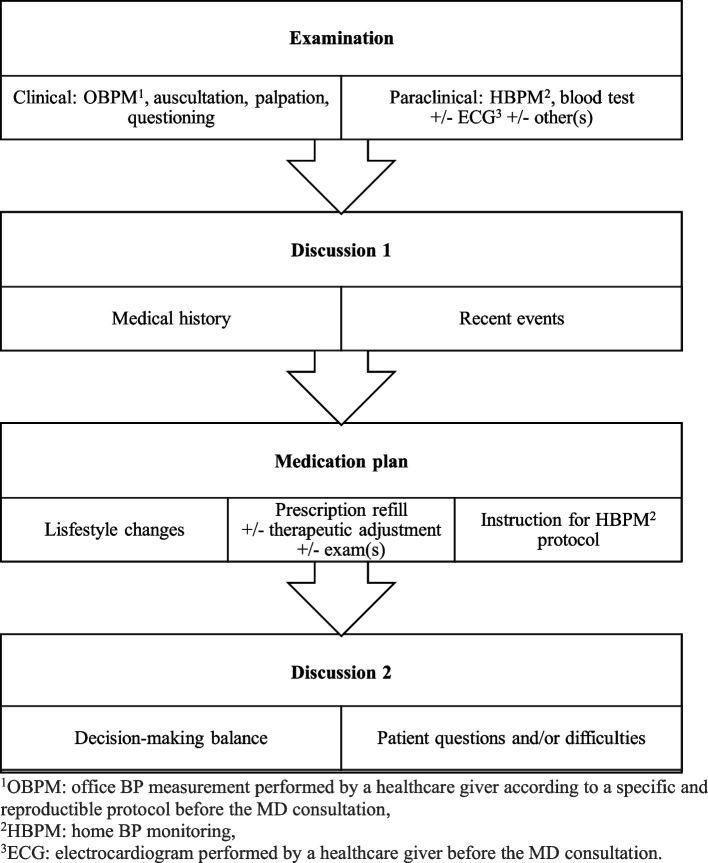
Plan for the MD consultation

The MD consultation will be divided into different stages: examination, discussions, and medication plan (Fig. 3).

Before the MD consultation, a healthcare giver (usually a nurse) will perform an office BP measurement according to the protocol described in the “Outcomes” section. An ECG will likewise be performed before the MD consultation.

The MD will proceed to the physical examination and evaluate drug treatments, tolerance, efficacy, and eventual modifications since the previous appointment.

He will check the home BP monitoring and other paraclinical exams such as ECG or blood test. While home BP monitoring is usually considered superior, according to its quality and taking into account potential masked or white coat hypertension, either the office BP measurement or home BP monitoring will be preferred by the MD to evaluate the BP level and the efficacy of the antihypertensive drugs taken during the study [1, 2].

The MD will carry out a summary of the medical history since the last follow-up, an assessment of the cardiovascular risk factors, and a clinical summary.

Finally, the MD will prescribe medication and paraclinical exams, if needed, for the next appointment.

If a participant does not show up for the MD consultation, we will call him/her to schedule another appointment in the limit of the data collection timeframe. If he/she still does not show up, the participant will be considered lost to follow-up.

### Data collection

All data will be collected on-site, none remotely.

The main criterion will be measured in the framework of a protocol detailed in the “Outcomes” section, supervised by a healthcare giver (usually a nurse), using an automatic sphygmomanometer that can print the statement of measures at the end of the monitoring.

As for the home BP monitoring, even if it is conducted by participants at home, data will be collected on-site during the MD consultation.

Information about therapeutic adjustments will also be collected on-site in medical files.

All information required by the protocol will be recorded on a case report form. An explanation will be provided for any missing data. The data will be collected as obtained and clearly transcribed on the case report form.

Erroneous data found on the case report form will be deleted and the corrected data will be copied, next to the deleted information, accompanied by the initials, the date, and any justification by the investigator or authorized person having made the correction.

The chief investigator and the coordinator will make the final decision to terminate the trial.

### Data monitoring committee

As the procedure cannot cause any adverse effects, no early termination is planned.

Data quality control on the case report form during statistical analysis will be performed to ensure that the data are complete, consistent, and plausible. In the event of an anomaly, the investigator will be asked to correct it. If a check is required in a source document, it can only be done by a member of the supervisory medical team.

An audit may be carried out at any time by persons designated by the sponsor and independent of those responsible for the research. The persons leading and monitoring the research agree to comply with the sponsor’s audit requirements. Verification can be applied at all stages of the research, from the development of the protocol to the publication of results and the classification of data used or produced in the research.

Any event resulting from a breach of protocol, standard operating procedures or applicable laws and regulations by an investigator, or any other person involved in the conduct of the research shall be subject to a no-action promoter compliance report (Assistance Publique – Hôpitaux de Paris). These non-conformities will be managed in accordance with the sponsor’s procedures.

The final report of the research with the human person referred to in Article R1123-67 of the CPMP shall be prepared and signed by the sponsor and the investigator. A summary of the report shall be prepared in accordance with the reference plan of the competent authority and shall be sent to the latter within one year of the end of the research, corresponding to the end of the participation of the last person having lent himself to the research.

### Statistical analysis

Data scientists will remain blinded by means of two databases:

- One database for all the participants with the data of the day hospitalization and the MD consultation (including the primary outcome). To maintain blinding, the groups will be called “group A” and “group B”
- One database with only the data of the APN intervention, so as to perform statistical analysis specific to this stage

The primary outcome measure (difference in rate of controlled BP between “intervention” and “usual care” groups) will be analyzed using a chi-square test. A *p* value < 0.05 will be considered significant. Participants with missing data for this primary outcome will be excluded.

For secondary outcome measures, the rate of performance and quality of the home BP monitoring between the “intervention” and “usual care” groups will be analyzed using a chi-square test. The difference between average BP at baseline and at follow-up will be determined for all the patients and separately for patients in the two study groups. Differences between baseline and follow-up will be tested for significance within paired *t*-tests within each group. Proportions of therapeutic adjustments and their indications between the “intervention” group and the “usual care” group will be tested for significance using a *t*-test for independent samples. For these secondary outcomes, missing data will be replaced by the mean and the standard deviation calculated on the sample, in case of quantitative variables, and neglected for the qualitative variables.

Non-adherent participants (APN intervention missed for the “intervention” group) will be considered in the statistical analysis: the rate of non-adherent participants will be mentioned in the results.

No adjustment for baseline differences is scheduled following the randomization. Nevertheless, statistical analysis may highlight participant characteristics, which we can correlate to controlled BP. If needed, an adjustment for baseline differences will be performed at a later time.

Correlations between the performance and quality of home BP monitoring in MD consultation and controlled BP will also be analyzed.

No interim analyses are scheduled.

Statistical analysis will be carried out using the R software.

## Discussion

### Progress to date

APNs are beginning to be established in the French healthcare system. This study aims to assess the impact of an APN intervention versus usual care on hypertension control in a timeframe of 2 to 12 months. It is innovative because it is the first in France.

Similar studies, implemented in other countries, have provided positive findings.

In 2014, the study by Dean et al. showed that patients followed up in a specialist nurse-led hypertension clinic had significantly greater systolic BP reduction compared to the usual care provided by general practitioners (144 vs 169 mmHg) [26].

Drevenhorn et al. in 2015 aimed to assess the self-care capacity of hypertensive patients and a possible correlation between their capacity to change their lifestyle with a personalized motivational interview carried out by a nurse as part of their comprehensive care. The motivational nursing interview improved the self-care capacity of these hypertensive patients, notably through the practice of physical activity [27].

Moreover, another trial in 2018 demonstrated that a personalized motivational interview of hypertensive patients carried out by advanced practice providers was associated with improved lifestyle and increased physical activity. It resulted in better BP management regardless of the patient’s cardiovascular risk evaluated by the Framingham risk score [28].

### Strengths of the study

Considering that the study will be conducted in a European center of excellence for hypertension, participants will benefit from the latest recommandations in hypertension management.

The centralized one-to-one randomization will ensure two equivalent groups are compared and shall limit selection bias.

The study will provide a good comparison between the current hypertension management in France and future management which will include APNs.

We will specify whether the reported drug adjustments were caused by side effects and/or non-control of hypertension, the objective being to emphasize the major role of APN interventions on medication. Assessment of the proportion of drug adjustments during the APN intervention and MD consultation will highlight the relevance of maximizing the frequency of (para)medical appointments in the global monitoring of hypertension.

### Limitations of the study

While the monocentric aspect of the study can constitute a limit, since the APNs’ arrival in 2020, there is only one APN working in a European center of excellence for hypertension. The fact that this study is specifically conducted in a European center of excellence for hypertension assures us of the quality of usual care in hypertension management. A multicentric study with other kinds of structures, which would not be European center(s) of excellence for hypertension, would generate many biases.

Even if home BP monitoring is considered superior to office BP measurement in usual care, office BP measurement over home BP monitoring for the primary outcome (rate of controlled BP) has been considered more relevant for the purposes of the study. Office BP measurement is systematic and conducted by health professionals according to a specific and reproducible protocol (identical in day hospitalization, APN intervention, and MD consultation) whereas home BP monitoring depends on participant involvement and ability.

The open-label character of this study could influence the reactions and behaviors of patients and healthcare givers and thereby introduce a performance bias.

The timing of the study could also be a limitation because health education for chronic diseases can take longer than a few consultations spread out over several months. A similar study over a longer period would likely better reflect the impact of APN intervention in global hypertension management.

This study will be carried out by a graduate APN. The level of experience of the professional involved in the study could create a bias. We may suppose that the results will vary according to the APN’s experience.

The results of the study will be interpreted according to these parameters and compared to the existing literature. It will be interesting to see if APNs can indeed represent a solution to a major public health problem consisting in uncontrolled hypertension in France.

## Data Availability

JVD, JB, and the statistician will have access to the final trial dataset. Confidentiality: To protect confidentiality, a pseudonym will be attributed to each participant, and data will be collected on a case report form and then transcribed onto an Excel table.
